# Involvement of shedding induced by ADAM17 on the nitric oxide pathway in hypertension

**DOI:** 10.3389/fmolb.2022.1032177

**Published:** 2022-10-14

**Authors:** Mirelly Cunha da Silva, Vanessa Maria dos Santos, Matheus Vinícius B. da Silva, Tereza Cristina M. M. Prazeres, Maria do Socorro S. Cartágenes, Natália Tabosa M. Calzerra, Thyago Moreira de Queiroz

**Affiliations:** ^1^ Laboratory of Nutrition, Physical Activity and Phenotypic Plasticity, Federal University of Pernambuco, Vitória de Santo Antão, Brazil; ^2^ Department of Physiological Sciences, Federal University of Maranhão, São Luis, Brazil; ^3^ Department of Physiology and Pharmacology, Federal University of Pernambuco, Recife, Brazil

**Keywords:** endotehlium, hypertension, inflammation, nitric oxide, metalloprotease, oxidative stress

## Abstract

A Disintegrin and Metalloprotease 17 (ADAM17), also called tumor necrosis factor-ɑ (TNF-ɑ) convertase (TACE), is a well-known protease involved in the sheddase of growth factors, chemokines and cytokines. ADAM17 is also enrolled in hypertension, especially by shedding of angiotensin converting enzyme type 2 (ACE2) leading to impairment of angiotensin 1–7 [Ang-(1–7)] production and injury in vasodilation, induction of renal damage and cardiac hypertrophy. Activation of Mas receptor (MasR) by binding of Ang-(1–7) induces an increase in the nitric oxide (NO) gaseous molecule, which is an essential factor of vascular homeostasis and blood pressure control. On the other hand, TNF-ɑ has demonstrated to stimulate a decrease in nitric oxide bioavailability, triggering a disrupt in endothelium-dependent vasorelaxation. In spite of the previous studies, little knowledge is available about the involvement of the metalloprotease 17 and the NO pathways. Here we will provide an overview of the role of ADAM17 and Its mechanisms implicated with the NO formation.

## Introduction

Hyperstimulation of the renin-angiotensin system (RAS), mainly associated with other cardiovascular risk factors, can induce a cascade of deleterious actions, such as an increase in blood pressure (BP) (Melaku, 2018). Hypertension is a multifactorial disease that results from the junction of genetic and environmental factors that present neuroendocrine, hemodynamic, redox and inflammatory components, which combine with each other to induce functional and structural changes in the cardiovascular system. (Prado et al., 2021).

The most active metabolite of the RAS is angiotensin II (Ang-II), which promotes vascular injury and hypertension primarily through an interaction with the Ang-II type 1 receptor (AT1R) (Yang et al., 2017). During RAS hyperactivity, there is evidence of increased activation of AT1R *via* Ang-II, which promotes translocation of ADAM17 to the cell membrane (Xu et al., 2017; Mukerjee et al., 2019). The ADAM family represents one of the main groups of sheddase proteases, which promotes proteolysis in the extracellular domain of integral membrane protein, controlling the biological activity of membrane proteins (Arribas and Borroto, 2002; Chow and Fernandez-Patron, 2007).

It has been described that ADAM-mediated cleavage may contribute to the tissue remodeling and dysfunction, as well as induction of inflammation. Thus, ADAM is related to the development of cardiovascular diseases (CVD), including atherosclerosis and hypertension, and may be a potential pharmacological target in the treatment of these diseases. (Kawai et al., 2021).

**FIGURE 1:**
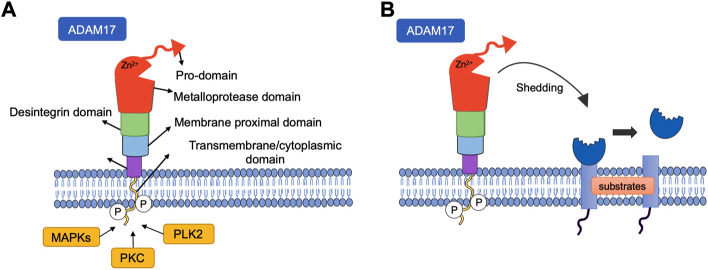
**(A)** Structure of ADAM metallopeptidase domain 17 (ADAM17). The structure of ADAM17 can be divided into six domains, all of which have distinct functions. CANDIS: Conserved ADAM-seventeen Dynamic Interaction Sequence. **(B)** Shedding of cell surface proteins induced by ADAM17. ADAM17 function is regulated by phosphorylation of the cytoplasmic domain by intracellular kinases such as protein kinase C (PKC), Polo-like kinase 2(PLK2), Mitogen Activated Protein Kinase (MAPKs). Various substrates, including receptors, cytokines and growth factors are targets of this protease, undergoing cleavage and release in the soluble form.

Among members of the ADAM family, ADAM17, also known as tumor necrosis factor (TNF)-α converting enzyme (TACE), acts as a metalloproteinase that cleaves the TNF-α precursor (Black et al., 1997; Kawai et al., 2021). Furthermore, ADAM17 also cleaves the active extracellular ectodomain of ACE2, releasing a soluble form of this enzyme (Figure 1), whose pathophysiology importance is not fully understood. (Melaku, 2018).

Membrane-bound ACE2 induces the production of Ang-(1–7) from Ang-II in vessel walls and other tissues. The Ang-(1–7) formed is involved in cardioprotective effects mediated by its binding and activation of the MasR (Yang et al., 2017), promoting the release of NO, decreased sympathetic activity and baroreflex dysfunction, as well as reduced BP levels (Xu et al., 2017). Thus, the ACE2-Ang-(1–7)-Mas axis counter-regulates the classic RAS in tissues involved in the maintenance of BP and homeostasis of the cardiovascular system (Rabelo et al., 2011).

ACE2 deficiency is associated with decreased NO bioavailability mediated downregulation of aortic endothelial nitric oxide synthase (NOS) expression (Rabelo et al., 2016). Furthermore, impairment of the ACE2/Ang-(1–7)/Mas pathway is associated with increased reactive oxygen species (ROS) production due to elevation nicotinamide adenine dinucleotide phosphate oxidase (NADPH oxidase) activity, as well as increased NO degradation (Rabelo et al., 2008). It has been shown that ADAM17 knockdown in the brain can restore ACE2 activity and attenuate the development of hypertension, suggesting that ADAM17-induced ACE2 cleavage is involved in the development of neurogenic hypertension (Xia et al., 2013). On the other hand, the inducible NOS (iNOS)/NO/cGMP/PKG pathway has also been shown to activate ADAM17, a mechanism that can limit inflammation (Chanthaphavong et al., 2012).

## The role of nitric oxide in hypertension

### Nitric oxide synthesis and regulation

NO is a free radical, gaseous, lipophilic, and highly reactive (Wu et al., 2021), that induces an important role in the regulation of vascular homeostasis (Cyr et al., 2020). Numerous evidences demonstrate that NO plays a central role in the maintenance of BP. (Ahmad et al., 2018a).

The gaseous molecule is synthesized by three distinct isoforms of the enzyme NOS, each with different functional properties and expression patterns: iNOS, neuronal NOS (nNOS) and endothelial NOS (eNOS). In general, these proteins are involved in the synthesis of NO and L-citrulline from oxygen (O_2_) and L-arginine. (Cyr et al., 2020). This enzymatic conversion also requires cofactors such as tetrahydrobiopterin (BH_4_) and NADPH (Ahmad et al., 2018a).

Although eNOS is found primarily in vascular endothelial cells, its expression has been shown in several other cells, such as renal tubular epithelium, platelets, cardiac myocytes and certain neurons in the brain (Cyr et al., 2020). Vasodilator agonists, such as acetylcholine and bradykinin, induce an increase in intracellular calcium and promote eNOS activation, increasing NO production in endothelial cells (Ahmad et al., 2018a). The NO formed in the endothelial cell diffuses into the smooth muscle cell, activating guanylate cyclase (sGC) and producing cyclic guanosine monophosphate (cGMP), which in turn activates protein kinase G (PKG) which mediates intracellular Ca^2+^ reduction, thus favoring smooth muscle relaxation (Reinero et al., 2021).

### The role of nitric oxide in vascular dysfunction

The positive regulation of NO/sGC/cGMP pathway in hypertension has been emerged as a promising therapeutic mechanism to drecrease BP (Ahmad et al., 2018a). Reduced bioavailability of NO contributes to vascular dysfunction in hypertension (Li et al., 2015; Wang et al., 2022). The term endothelial dysfunction (ED) is a disorder characterized, anatomically, by intact endothelial cells (Di Daniele et al., 2021), but with alteration in the production of endothelium-derived factors, such as increased synthesis of ROS, abnormal production of pro-inflammatory cytokines, like TNF-α and interleukin-1 (IL-1), increased production of cyclooxygenase (COX) derivatives, such as thromboxane A_2_ and prostaglandins H2, E2, and F2α (Félétou, 2009; Bernatova, 2014; Kakabadze et al., 2021). In addition to attenuation of endothelium-derived hyperpolarizing factor and decreased production and action of NO.

In general, decreased NO bioavailability in hypertension is associated with decreased synthesis by eNOS or increased inactivation of NO by oxidative stress (Pinheiro et al., 2017). One study has showed a reduced activity and expression of eNOS in spontaneously hypertensive rats (SHR) (from 4 to 14 weeks), but not in normotensive Wistar Kyoto (WKY) control, which may contribute to the development of hypertension (Chou et al., 1998). Furthermore, in this genetic model of hypertension, it was also shown that hyperactivity of NADPH oxidase and the superoxide (O_2_^-^) produced are associated with reduced NO-dependent relaxation, ED, and vascular hypertrophy (Zalba et al., 2000).

In some disease conditions, including hypertension, activation of NADPH oxidase, especially NOX1 and NOX2 isoforms, promotes the synthesis of ROS, which causes the oxidation of BH4 to BH_2_, resulting in eNOS uncoupling (Li et al., 2015; Wu et al., 2021). This uncoupling is characterized by the discrepancy between NO production and eNOS levels, due to changes in the action of this enzyme, with consequent production of the O_2_^-^ ion instead of NO (Bouloumié et al., 1997; Montezano and Touyz, 2012; Yuyun et al., 2018).

### Nitric oxide and inflammation

Certain cytokines have been reported to act on endothelial cells, contributing to reduced production and activity of vasodilator mediators, such as NO (Sprague and Khalil, 2009). As cells of the immune system are activated in hypertension, they produce cytokines which act on the adjacent tissue to promote the synthesis of ROS derived from NADPH oxidase (NOX1 and NOX2) (Manea, 2010). In the vasculature, for example, cytokine-stimulated ROS synthesis induces NO inactivation and attenuation of endothelium-dependent relaxation (Griendling et al., 2021).

It has been reported an increase in arginase expression in endothelial cells and acute inflammatory cells, with consequent reduction in L-arginine concentration, the substrate of eNOS (Cyr et al., 2020). Furthermore, TNF-α has also been shown to decrease eNOS mRNA levels in human endothelial cells (Yoshizumi et al., 1993).

TNF-α has already been shown to stimulate ROS production in endothelial cells, smooth muscle cells, and neutrophils (Madge and Pober, 2001; Sprague and Khalil, 2009). On the other hand, the ROS formed activates transcription factors, such as nuclear factor Kappa-B (NF-κB), which will modulate the gene expression of adhesion molecules, chemokines, and pro-inflammatory cytokines (Theofilis et al., 2021), that induce the activation and infiltration of more immune cells, such as macrophages, contributing to target organ damage, characteristic of hypertension (Griendling et al., 2021).

The action of cytokines, such as IL-1β, on macrophages stimulates the production of large amounts of NO by iNOS (Szabo, 1995). Increased expression of iNOS in SHR cultured smooth muscle cells has also been demonstrated (Wu et al., 1996). Large volumes of NO react with O_2_^−^ to produce peroxynitrite, which can inhibit the activity of eNOS and sGC (Xia and Zweier, 1997; Leo et al., 2015).

It has been shown that the production of NO by iNOS has an effective role in the exacerbation of inflammation, which can promote an increase in leukocyte cytotoxicity during the inflammatory process, and also in the regulation and increase of COX-2, which is the enzyme that produces the mediators of inflammation (Hunter, 2002; Ye et al., 2008).

### Nitric oxide interventions in hypertension

Blocking the NO production pathway using pharmacological tools such as the NOS inhibitor NG-nitro-L-arginine methyl-ester (L-NAME) has been used to study the importance of NO in hypertension (Ahmad et al., 2018a). Some studies demonstrate that inhibition of NO synthesis through oral administration of L-NAME produces arterial hypertension (Baylis et al., 1992; Seth et al., 2016), as well as cardiac remodeling, such as left ventricular hypertrophy and myocardial fibrosis, in addition to aortic wall thickening and aortic collagen deposition (Bunbupha et al., 2015). Corroborating previous reports, Jin et al. (2017) also demonstrated that L-NAME promoted cardiac dysfunction accompanied by reduced eNOS expression and NO bioavailability (Jin et al., 2017).

An important mechanism of L-NAME-induced hypertension is oxidative stress, since in this model the uncoupling of eNOS is evidenced, leading to an overwhelming generation of vascular O_2_^-^ (Maneesai et al., 2018). According to Boonprom et al. (2017), the molecular mechanism related to cardiac fibrosis of L-NAME hypertensive rats involves the activation of oxidative stress (Boonprom et al., 2017). It has already been described that the lipid peroxidation was elevated in L-NAME-induced hypertension (Kumar et al., 2010; Kumar et al., 2012). In addition, significantly decreased levels of catalase (Cat), glutathione peroxidase (GPx) and superoxide dismutase (SOD) were evidenced in cardiac and aortic tissue (Sawant and Bodhankar, 2016) and erythrocytes (Kumar et al., 2012) of rats with L-NAME hypertension (Bunbupha et al., 2015).

Pro-inflammatory phenotypic alterations are also described in hypertension induced by NOS inhibition by L-NAME, including increased expression of vascular cell adhesion molecule-1 (VCAM-1) and intercellular adhesion molecule-1 (ICAM-1) in the vascular wall (Luvarà et al., 1998). Elevated transforming growth factor beta 1 (TGF-β1) plasma levels and renal TGF-β1 expression have also been reported, indicating that the raise in TGP-β1 expression may be related to renal and vascular changes induced by NOS suppression in the L-NAME model (Bunbupha et al., 2021). In this same model, increases in cardiac expression and serum levels of cytokines, such as interleukin-1 beta (IL-1β), interleukin-6 (IL-6), and TNF-α were also demonstrated (Miguel-Carrasco et al., 2008; Bunbupha et al., 2015), as well as the overexpression in vascular and cardiac tissue of the protein iNOS (Bunbupha et al., 2015; Leo et al., 2015).

RAS overactivation may be involved in vascular and renal dysfunction, mediated by hypertension induced by NO biosynthesis inhibition. These abnormalities have been associated with increased serum angiotensin converting enzyme (ACE) activity as well as upregulation of AT1R expression in the kidney (Bunbupha et al., 2021). Research has shown increased ACE activity in both heart and aortic tissue in L-NAME hypertension, demonstrating a relationship between ACE activity and NO synthesis in this model of hypertension (Sawant and Bodhankar, 2016).

In addition, L-NAME administration increases Ang-II levels and promotes cardiac AT1R overexpression, and these effects are associated with left ventricular wall hypertrophy and fibrosis (Sonoda et al., 2017; Gao et al., 2018). In addition, exogenous Ang-II has also been documented to reduce L-arginine transport in aortic endothelial and renal cells of mice, which in turn can induce a reduction in NO availability and support the hypertension development (Rajapakse et al., 2014).

**FIGURE 2:**
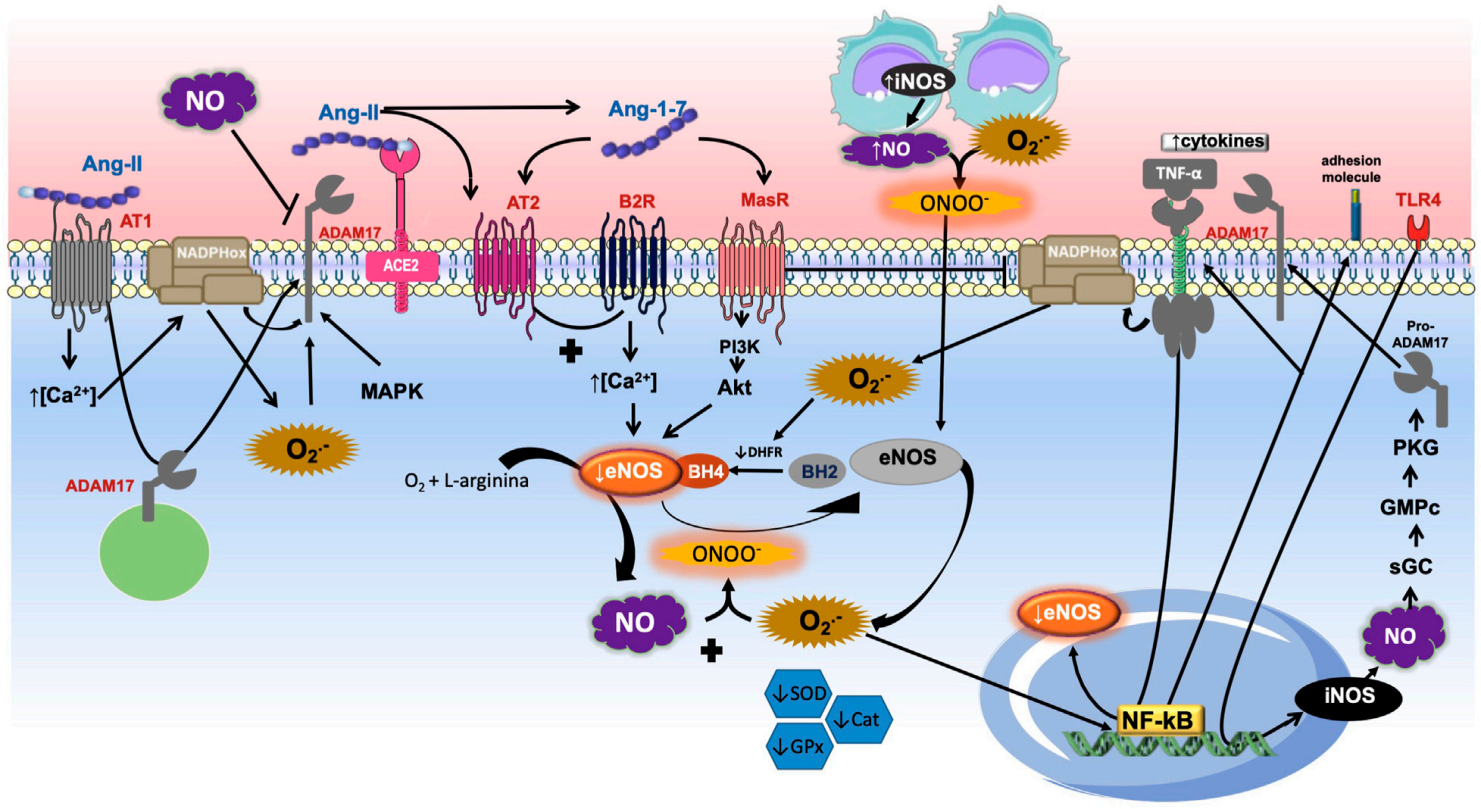
Role of ADAM17-mediated shedding and nitric oxide (NO). The Angiotensin-II (Ang-II) binding into Ang-II type 1 receptor (AT_1_R) induces the calcium concentration increase, which promotes nicotinamide adenine dinucleotide phosphate oxidase (NADPH oxidase) stimulation and increases the production of superoxide anion (O_2_^-^), which interacts with NO to produce peroxynitrite. Reactive species oxidize BH4 to BH2 causing eNOS uncoupling. The binding of Ang-II into its receptor causes desintegrin and metalloprotease 17 (ADAM17) moving to the membrane and its activation directly. ADAM17 also can be activated by reactive oxygen species (ROS) and MAP kinase family (MAPK). Once the ADAM17 is in the cell surface it induces shedding of diverse membrane-anchored proteins such as the angiotensin converting enzyme type 2 (ACE2), inhibiting the conversion of Ang-II into Ang-(1–7). The heptapeptide binds into Mas receptor (MasR) which induces PI3K/AKT pathway stimulation and NO formation through endothelial nitric oxide synthase (eNOS). Similarly, Ang-(1–7) is able to promote Ang-II type 2 (AT2R) receptor activation, which stimulates the bradykinin (BK)-NO cascade by bradykinin type 2 receptor (B2R). The increase of inducible NOS (iNOS) promotes NO production, which in the presence of high concentrations of O_2_^-^ induces peroxynitrite (ONOO^−^) formation, further contributing to the uncoupling of eNOS. In addition, decreased levels of the endogenous antioxidants superoxide dismutase (SOD), glutathione peroxidase (GPx), and catalase (Cat) were observed. The pro-inflammatory binding of tumor necrosis factor *α* (TNF-α) into its receptor stimulates NADPH oxidase and ROS formation. O_2_^-^ induces nuclear factor Kappa-B (NF-κB) promotion and decreasing eNOS, activation of adhesion molecules and enhance of TNF-α pathway. This figure also shows that the activation of toll-like receptor 4 (TLR4) stimulates the transcription of iNOS and consequent activation of guanylate cyclase (sGC), cyclic guanosine monophosphate (cGMP), protein kinase G (PKG) pathway. It promotes the maturation and translocation of ADAM17 to cell surface, with consequent shedding of TNF-α receptor and decreasing of inflammation development.

As previously described, NO is involved in several functions, such as regulating BP, vascular tone, smooth muscle proliferation as well as being involved in the inflammatory process (Machha and Schechter, 2011; Shreshtha et al., 2018). An important source of NO in the cardiovascular system is eNOS, but a variety of endogenous and exogenous stressors can alter the production of NO, in addition to increase its degradation (Cyr et al., 2020). These stressors, such as RAS overactivation, oxidative stress and inflammation, contribute to an imbalance that favors an increase of ROS and consequent reduction in NO bioavailability, which promotes endothelial dysfunction and increase in blood pressure (Figure 2).

## Role of ADAM17-induced shedding and inflammation in hypertension

ADAM17 posses a great dissemination and It is involved acting as a sheddase by proteolytic cleavage of several cell surface proteins enroled in the inflammation progression, involving proteins of the signal transduction, control of cell adhesion, and release of growth factors and various cytokines (Hao et al., 2004; Garton et al., 2006; Iwata et al., 2009). The TNF-ɑ, cytokine receptors (IL-6 receptor (IL-6R), macrophage colony stimulating factor I (M-CSFRI) and TNF-receptor I and II (TNF-RI and TNF-RII) are among the most studied cytokines/receptors as targets for ADAM17 shedding (Müllberg et al., 1993; Black et al., 1997; Garton et al., 2001; De Queiroz et al., 2020).

TNF-ɑ has pro-inflammatory effects which are related with activation of TNFR1, and it increased in human serum was associated to be a signal in the hypertension development in patients with chronic kidney disease (Saulnier et al., 2014). The deletion of TNFR1 gen in mice led to a rise in systolic BP in response to Ang-II infusion (Chen et al., 2010). Another study demonstrated that deletion of TNFR1 in subfornical organ ameliorates sympathetic drive and heart failure in rats (Yu et al., 2017), which can be due to inhibition of TNFR2 shedding, leading to TNF-ɑ actions and increasing BP induced by Ang-II (Chen et al., 2010). All together, these findings indicate that selective activation or deletion of TNFR1 contributes to a mechanism that may lower or increase BP, respectively.

Studies have shown that the ADAM17-dependent TNF-ɑ shedding involves a critical molecule called inactive rhomboid protein 2 (RHBDF2, known as iRhom2). This protein is included in the family of rhomboid protease of catalytically inactive serine proteases. In mammals, two iRhoms were identified, iRhom1 and iRhom2. The first one is ubiquitously expressed in many cells and tissues, whereas iRhom2 is assumed to be expressed mostly in cells of immune system (Babendreyer et al., 2020). This protein is enrolled in ADAM17 maturation (Siggs et al., 2012), however the underlying mechanism is still little explored (Li et al., 2017).

The metalloprotease 17 is synthesized with a pro-form with a prevention of the catalytical domain of the sheddase. Once ADAM17 is carried from the endoplasmic reticulum (ER) to the Golgi complex (GC), the pro-domain is cleaved by pro-protein convertases, such as furin. Then, the proteolytically active, mature ADAM17 is carried to the cell surface (Endres et al., 2003). iRhoms are key controllers in this mechanism likewise they are crucial for the transport of ADAM17 from the ER to the GC (Adrain et al., 2012; Mcilwain et al., 2012; Babendreyer et al., 2020).

Alterations evoked by ADAM17-induced cytokines shedding and other membrane proteins, such as ACE2, may lead to endothelium dysfunction (Kawai et al., 2021), which in turn, induces vascular hemodynamics, endothelial damage, barrier dysfunction, and dysregulation of vascular tonus control, thus changing the vascular environment, leading to cardiovascular incidents (Zhang et al., 1995; Zhang et al., 2020; Da Silva et al., 2021).

The blood flow applies a continuous shear stress on the endothelium. Biological levels of shear stress led to a vasorelaxant, anti-thrombotic and anti-inflammatory phenotype in endothelial cells. ED is associated with an inflammatory response of the endothelium (Rajendran et al., 2013). Inflammatory responses may be beneficial to remove pathogens in the tissue or to initiate its repair. Nevertheless, persistent inflammation provides ED, which can produce various CVDs (Davignon and Ganz, 2004).

The relation between inflammation and hypertension have been studied for more than two decades, however the precise pathophysiological mechanism involved in that association is intricate and partly understood.

The activation of bradykinin receptor B1 (B1R)-mediated signaling pathways, a very known peptide inductor of inflammation, is associated in the pathogenesis of many CVDs that are associated with or induced by inflammation (Sriramula, 2020). Studies have showed that activation of B1R in the PVN occasioned elevated neuroinflammation, ROS production and sympathoexcitation, inducing to the development of neurogenic hypertension in deoxycorticosterone acetate-salt (DOCA)-treated mice (Sriramula and Lazartigues, 2017). In addition, our collaborators have demonstrated the evidence for ADAM17-mediated ACE2 shedding in neurons (Xu et al., 2018). For the first time, Parekh and Sriramula (2020) have showed that B1R activation produced an ADAM17-mediated shedding and lower ACE2 activity in neurons. Furthermore, the results support the new concept that B1R is implicated in glutamate-mediated effects on ADAM17 activity and cleavage of ACE2 (Parekh and Sriramula, 2020).

In an interesting study using a mouse model of Ang-II-induced hypertension without smooth muscle ADAM17 participation, caused by gen deletion or systemic pharmacological inhibition of ADAM17, the vascular hypertrophy and perivascular fibrosis were reduced (Takayanagi et al., 2016). The mechanism involved in this effect has been associated to the mediation of ADAM17 in the epidermal growth factor receptor (EGFR) transactivation induced by Ang-II in vascular smooth muscle cells (Forrester et al., 2016; Kawai et al., 2021). Furthermore, *in vitro* findings have showed that ROS are also able to induce the activation of ADAM17 in platelets, and this effect would illustrate a limiting mechanism for platelet function (Brill et al., 2009). Also, the production of ROS mediated by NADPH oxidase 4 (NOX4) is required to induce ADAM17 expression and following induction of cardiac hypertrophy (Zeng et al., 2013).

Our research group has used an antioxidant approach with the lipoic acid (LA) to demonstrate that LA was able to reduce BP and improves baroreflex sensitivity in renovascular hypertensive rats (Queiroz et al., 2012). In addition, using Neuro2A cells (neuroblastoma cell line) and a DOCA-salt model of hypertension, the antioxidant therapy preserved ACE2 compensatory role by breaking the feedforward cycle between oxidative stress and ADAM17 especially due to the oxidative stress decreasing. Thus, ADAM17 could be a new target to preclude the development of neurogenic hypertension (De Queiroz et al., 2015). Moreover Zeng et al. (2019) documented that NADPH oxidase, such as Nox1/4 subunits inhibitor GKT137831 inhibited hypertensive cardiac remodeling, and it reduced the expression of ADAM17 and pro-inflammatory cytokines such as TNF-α, IL-1β and IL-6 in abdominal artery coarctation-induced hypertensive rats (Zeng et al., 2019).

The findings described in this topic demonstrate the critical role of ADAM17. This metalloprotease needs to be in the active form to induce the shedding of multiple proteins related to inflammation such as cytokines, adhesion molecules and cytokines receptors. The activation and transportation of ADAM17 to the membrane surface is elicited by iRhom proteins, especially iRhom2. In addition, the cytokine TNF-ɑ is recognized to promote inflammation and BP increasing by its binding in TNF-ɑ receptors. On the other hand, It is already known that brain or vascular inflammation promotes increase of BP. This mechanism involves ROS production, ADAM17 activation and ACE2 dysfunction with a injury in Ang-(1–7) formation and deleterious effects, including hypertension development.

## Role of ADAM17-induced shedding and nitric oxide in hypertension

In hypertension induced by chronic inhibition of NO synthesis, it has been described that Ang-(1–7) can attenuate BP elevation and target organ damage in L-NAME-treated SHR (Benter et al., 2006). It was also described that activation of the MasR by Ang-(1–7) causes a vasorelaxant effect through the eNOS-NO-cGMP-PKG pathway, which contributes to the decrease in BP in SHR (Zhang et al., 2019). However, ACE2, the Ang-(1–7) producing enzyme, deficiency is associated with lower NO bioavailability through a reduction in aortic eNOS expression (Rabelo et al., 2016).

In addition, the vasoprotective effects of Ang-(1–7) are also related to the maintenance of NO availability due to the reduction in ROS (Heitsch et al., 2001). Benter et al. (2008) observed that Ang-(1–7) promotes inhibition of NADPH oxidase NOX4 and prevents renal vascular dysfunction in diabetic hypertensive rats (Benter et al., 2008). Another study documented that chronic infusion of Ang-(1–7) attenuated the increase in the expression of gp91phox, a catalytic subunit of the NADPH oxidase, in a dose-dependent manner in SHR brain (Jiang et al., 2013). Moreover, indicators of oxidative stress were evidenced in both the aorta and plasma of mice with MasR gene deletion, this was demonstrated by the higher levels of thiobarbituric acid reactive substances, upregulation of gp91phox and reduction in SOD and catalase activities in this animal model (Xu et al., 2008).

It has also been reported that reduced levels of NO can reduce the formation of Ang-(1–7) (Rajapakse et al., 2019). Interestingly, one study suggests that NO is a permissive factor for macula densa renin release (Castrop et al., 2004) and that exogenous (NO donor) and endogenous NO inhibit the ACE from human serum and cultured endothelial cells (Persson et al., 2000). Based on these results, Rajapakse et al. (2019) suggested that NO can act through activating a cardioprotective pathway of the RAS, by increasing the production of Ang-I, but reducing the conversion of Ang-I to Ang-II. In this sense, more Ang-I may be available for the synthesis of Ang-(1–7) (Rajapakse et al., 2019). In addition, studies have showed that NO directly interacts with AT1R, promoting its inhibition as showed in vascular smooth muscle cells (Ichiki et al., 1998; Ahmad et al., 2018a). Also, the increase of the exogenous NO precursor induced an upregulation in the eNOS/NO/cGMP pathway and diminished the Ang-II levels in a model of cardiac hypertrophy in rats (Ahmad et al., 2018b).

A correlation between NO and ADAM17 has been documented, as it was evidenced in research that exogenous NO donor reduces ADAM17 activity, as shown by the decrease of substrates released by ADAM-17 in murine endothelial cells (Bzowska et al., 2009). This reduction in NO-induced ADAM17 activation possibly involves nitrosylation of the thiol group on cysteine residues in the inhibitory prodomain of this enzyme (Zhang et al., 2000; Xu et al., 2016).

The mechanisms of ADAM17 activation still remain controversial, however, some evidence points to ROS, NOX4 and MAP-kinase family proteins as possible activators (Brill et al., 2009; Kuan et al., 2013; Zeng et al., 2013). Some years ago, NO was revealed as an activator of TACE, as shown by Chanthaphavong et al. (2012) that demonstrated the nitric oxide synthase (iNOS) stimulating factors such as lipopolysaccharide (LPS), through the Toll-like four receptor (TLR4), induce iNOS transcription, which culminates in the production of NO in hepatocytes. This lipid-soluble molecule binds to sGC which promotes the formation of cGMP and activation of PKG, which in turn promotes the phosphorylation and activation of ADAM17 (Chanthaphavong et al., 2012).

NO also plays an important role in activating ADAM17. Studies have showed that increases in Ca^2+^ concentrations can also induce NOS activation and promote NO release, which has the ability to inhibit the cytochrome oxidase enzyme of the electron transport chain and ultimately leads to formation of ROS, activating ADAM17 (Dada and Sznajder, 2011).

The activation of ADAM17 is related with the up-regulation of iRhom2, likewise the phosphorylation of ADAM17 and iRhom2, which are also dependent of NO/cGMP/PKG pathway. These conclusions indicate that the increase of iNOS/NO expression includes the rapid shedding of TNFR1 to limit the TNF-ɑ signaling (Chanthaphavong et al., 2012; Deng et al., 2015). Due to a central and tissue specific regulator of ADAM17, recently iRhom2 has arisen as a novel target protein for a specific inhibition of ADAM17 (Geesala et al., 2020).

TNF-ɑ signaling also has been associated with a growth of salt appetite and sodium reabsorption stimulation in renal tubules by a mechanism that involve the suppressing of NOS, becoming a critical factor to induce hypertension (Ramseyer and Garvin, 2013; Zhang et al., 2014; Rudemiller and Crowley, 2016). In addition, TNF-ɑ knockout mice showed a growth in eNOS formation and prevented an increase in BP of Ang-II-induced hypertension mice in comparison with the wild type animals (Sriramula et al., 2008; Rodriguez-Iturbe et al., 2017).

In addition to NO, other endogenous gaseous mediators have revealed to modulate ADAM17 level/expression, such as the gas hydrogen sulfide (H_2_S). Studies have showed that H_2_S eliminates the both mRNA and protein TNF-α-induced TACE expression. H_2_S was able to prevent TNF-α-induced endothelial damage through a protective mechanism mainly mediated by downregulation of ADAM17 activity with following suppression of soluble TNF-α shedding and the cytokine monocyte chemoattractant protein (MCP-1) release in the endothelial cells medium. These findings show the protective role of H_2_S, especially as inhibitor of inflammatory and pro-atherogenic processes (Perna et al., 2013).

As mentioned earlier, NO is involved in diverse physiological mechanisms, including the pathways that promote BP reduction. NO releasing can be evoked by MasR activation induced by Ang-(1–7), which is formed, especially, by ACE2, inducing in turn, reduction in BP. This convertase enzyme could be negatively modulated by ADAM17. Studies have demonstrated that NO could inhibit ACE expression and also ADAM17 activity. Conversely, ROS, NOX4 and other MAP kinases were able to activate ADAM17, in addition to Ang-II binding into AT1R. Furthermore, NO produced by iNOS would induce the cGMP/PKG pathway, promoting ADAM17 phosphorylation and its activation. The active form of ADAM17 is able to induce TNFR1 shedding and limit the TNF-ɑ actions in that receptor. Nevertheless, these actions persist controversial (Figure 2).

## Future directions of ADAM17 as a therapeutic target in hypertension

ADAM17 orchestrates many different signaling pathways linked to hypertension and other CVDs as showed in this review. ADAM17 has showed to induce neointimal hyperplasia in vasculature (Takaguri et al., 2011). The TACE expression is increased in atherosclerosis (Canault et al., 2006) and in the left ventricle after Ang-II infusion (Patel et al., 2014) and that ADAM17 polymorphism is associated with cardiovascular mortality (Morange et al., 2008; Takayanagi et al., 2016). Therefore, the metalloprotease 17 presents itself as a possible therapeutic target to the treatment of hypertension.

Ludwig et al. (2005) identified an ADAM17 inhibitor, named GW280264X, which has showed to block the constitutive release of mIL-6R, CX3CL1/fractalkine, and chemokine C-X-C ligand 16 (Ludwig et al., 2005). Recently, another study has developed a new ADAM17 inhibitor composed of a zinc-binding dithiol moiety, SN-4, which showed a specif binding to ADAM17, avoiding TNF-α shedding (Tateishi et al., 2021). Together, the studies demonstrate possible future TACE inhibitors. These effects would induce a decrease in inflammation and a consequent improvement in hypertension. In addition, inhibition of other ADAM17 activators for instance ROS, as showed by our group using lipoic acid or a SOD mimetic, tempol, were able to prevent the hypertension development in a DOCA-salt hypertension model (De Queiroz et al., 2015).

ADAM17 also triggers BP through a CNS-dependent mechanism. DOCA-salt hypertensive rats exhibited an ADAM17 increase in the hypothalamus, with a consequent reduction in ACE2 expression and activity in the brain, inducing a BP enhance, inflammation and autonomic dysfunction. So, knockdown of ADAM17 in the brain can blunt the development of hypertension (Xia et al., 2013). Furthermore, in the brain of hypertensive patients ADAM17-mediated ACE2 shedding seems to be stimulated by Ang-II, suggesting the participation of ADAM17 in neurogenic hypertension in human (Xu et al., 2017; Kawai et al., 2021).

Studies also have showed limitations to reveal the ADAM17 as a therapeutic target in hypertension. The increase of ADAM17 showed an essential role in the signal transduction for cardiovascular remodeling associated with ER stress however not for hypertension in Ang-II-treated mice (Takayanagi et al., 2016). Other findings have demonstrated that TNF-ɑ has associated with reduction in BP and strong inflammation. These opposite effects probably are due to the biding into two different receptors TNFR1 and TNFR2. Thus, the exact receptor subtype in ADAM17 downstream pathway to induce TNF-ɑ shedding still controversial (De Queiroz et al., 2020).

As shown along this manuscript, the protein iRhom2 acts as a key molecule in the ADAM17 activation and transportation to cell surface membrane. For this reason, the inactive rhomboid protein also becomes a possible pharmacological target to the treatment of hypertension, however more studies are need to address this question.

## Conclusion

Here we briefly reviewed the role of ADAM17 shedding and nitric oxide. Both metalloprotease and gaseous molecule have showed central roles in the hypertension increase and promotion of other cardiovascular diseases. NO is the main gaseous molecule released from the vascular endothelium, which induces vasodilation mainly by NO/sGC/cGMP/PKG pathway. ADAM17, also named TACE, is involved in the shedding of many inflammatory cytokines and also in ACE2 cleavage. This last peptide is critical in the conversion of Ang-II into Ang-(1–7), which promotes the anti-hypertensive effects through the binding into MasR or AT2R, equally inducing NO releasing. ADAM17 can be activated by ROS, NOX4 and MAP-kinase family proteins. Furthermore, the activation of ADAM17 is associated with the up-regulation of iRhom2, which is phosphorylated by a NO/cGMP/PKG-dependent mechanism. This last mechanism was observed in hepatocytes, which suggest us to study it in a cardiovascular model of hypertension. Therefore, once ADAM17 is expressed in the membrane, it stimulates the shedding and pro-hypertensive effects. However, the ADAM17 inhibition by ROS decreasing, downregulation of some MAP-kinases, or upregulation of Ang-(1–7)/MasR pathway, inducing NO formation, produces reduction in hypertension development.
